# New York Academy of Medicine

**Published:** 1885-04

**Authors:** 


### Society Reports.

### NEW YORK ACADEMY OF MEDICINE.*

STATED MEETING, February 19, 1885.

A. Jacobi, M. D., President, in the chair.

### ELECTRICITY AS A STIMULANT IN CARDIAC AND RESPIRATORY FAILURE.

Dr. Gaspar Griswold read a paper on the above subject, beginning with the statement that electricity had been conspicuous in the treatment of sudden prostration, attended with respiratory and cardiac failure, especially in chloroform inhalation and opium-poisoning. The object of the paper was to discuss how far the usual methods of applying electricity in such cases of collapse were in accord with what was known concerning the physiology of the heart. The term electricity was used in a general sense, including galvanism and faradism.

Can electricity be so applied as to stimulate the heart? Prefatory to the answer to this question the author of the paper gave a brief synopsis of the cardiac mechanism, directing special attention to the effect produced by stimulating the pneumogastrics. From the study of this mechanism the conclusion was reached that, to stimulate the heart, the electrical current must be applied directly to the heart itself, and that the application of the poles to the neck over the pneumogastrics must be to produce an effect the reverse of stimulation. The question must, therefore, be answered in the negative.

Dr. Griswold then entered into the study of the effect produced when electricity was applied to the phrenic nerves, and reached the conclusion that electricity applied to the phrenic nerve stimulates respiratory movements.

*The Medical Record, February 28th.

On physiological and clinical grounds, therefore, stimulation of the phrenic nerve by itself was a proper and logical treatment in respiratory failure, but stimulation of the phrenic nerve could be scarcely considered separately from stimulation of the pneumogastrics, because these nerves were situated so closely to each other in the neck. In other words, electricity could not be applied to the phrenic nerve to stimulate contractions of the diaphragm without great danger of retarding or cutting off the function of the pneumogastric.

The conclusions, therefore, of the author of the paper, were as follows: 1. Electrical stimulation of the phrenic nerve should not be undertaken without the reflection that the current will at the same time reach the pneumogastric. 2. Only mild currents should be applied, and their effects carefully watched, but especially with reference to the heart. 3. The sudden and careless application to the neck of a current sufficiently strong to produce pain or muscular contractions in other parts of the body, should be especially avoided.

Dr. Griswold then gave a detailed description of experiments which he had performed in dogs with special reference to the effects produced on the pneumogastrics, both by electricity and by drugs administered in poisonous doses.

### ACONITE-POISONING.

The stimulant effect of electricity upon the pneumogastric in these cases did no harm, because aconite in poisonous doses produces paralysis of the pneumogastric. The application of electricity, therefore, to the phrenic nerve with proper care, in aconite-poisoning, was perfectly safe.

### CHLOROFORM-POISONING.

Stimulating the pneumogastric caused fatal results at once. When heart-failure occurred early in chloroform administration, the pneumogastrics were still normally excitable, and at such times a very mild electrical current would instantly paralyze them and fatally depress the action of the heart. Therefore, it was immensely dangerous to apply electricity to the neck of a person poisoned with chloroform, and also when applied over the phrenic nerve it would almost certainly cause a fatal termination.

ASPHYXIA FROM ETHER.

In this condition the heart could stand stimulation as well as in health, and, therefore, it was safe to stimulate it with the strongest electrical current which the patient could bear in health.

Chloroform was a direct cardiac depressant; ether did not depress the heart.

OPIUM-POISONING.

Morphine in poisonous doses paralyzes the pneumogastrics, and stimulation of them by electricity would not slow or depress the action of the heart.

In opium-poisoning, when the action of the heart was rapid and feeble, there was less danger than in health of cardiac paralysis from stimulation of the pneumogastrics. When

MORPHINE WAS INJECTED INTO THE VEINS

the heart was easily depressed by electricity applied to the pneumogastrics, and it would not, therefore, be safe to faradize or galvanize the phrenics in this condition, because of the certainty, almost, of stimulating at the same time the pneumogastrics.

A general conclusion reached by the author of the paper was that, under no circumstances, should an electrical current, sufficiently strong to produce contractions of the muscles in any part of the body, be applied over the phrenic or pneumogastric in the neck.

Dr. A. D. Rockwell, in general, quite agreed with the statements made by Dr. Griswold, in his valuable contribution to the literature of this subject. The fact that, in the normal condition, and by purely external methods of application, the respiration was affected, rather than the heart's action, was very readily demonstrated. In connection with this subject, he might have referred to the statements made long ago by Arloing and Tripier, that the right pneumogastric had a more powerful influence over the heart, while the left more powerfully affected respiration. However that may be, it was certain that galvanization affected the two pneumogastric nerves unequally, and in a way to confirm, to some extent, that assertion.

It had been found that, if the heart be stopped by galvanization of the exposed left pneumogastric, the movements could be restored by some slight mechanical excitation, while, if the same result occurred through galvanization of the right pneumogastric, it seemed impossible to again excite the pulsations. These, certainly, seemed to be the effects of sedation rather than of stimulation, but that the effects that followed were powerfully stimulant and tonic could not be doubted.

Some years ago, in a number of experimental attempts in the electrolytic treatment of cancer, Dr. Rockwell had occasion to observe some interesting phenomena in regard to the primary and secondary effects of the galvanic current on the heart's action. In cases of scirrhus, after the breast had been removed by the knife, it was his custom to destroy, as thoroughly as possible, the underlying tissue, by the electrolytic method. It was readily understood that the effects of this concentration of the current through needles penetrating the muscular tissue were much greater than any ordinary applications by means of sponges. The heart would slow up according to the tension of the current, and would sometimes become so alarmingly slow as to necessitate a cessation of the current for a time. The interesting point which was observed and commented upon by every physician who aided in, or was present at, those operations, was the fact that, following the breaking of the current, the heart's action became unmistakably more vigorous than at any previous time.

According to Professor Von Ziemssen, who made many carefully-conducted experiments by means of cardiographic tracings, induced currents have absolutely no influence on the frequency or force of the cardiac contractions, and from that fact the conclusion had been drawn that induction currents were valueless in threatening death from heart-failure. Dr. Rockwell's experience did not confirm that statement, for, in not a few instances of the kind, he had known life to be saved by this form of electricity. The faradic current, by its power over the phrenic nerve, excited respiration, and so indirectly very decidedly influenced the action of the heart itself. He certainly agreed with Dr. Griswold in the necessity of caution in applications of galvanism to the region of the phrenic and pneumogastric, and it was better, perhaps, to err on the side of safety, yet Dr. Rockwell could not but think that Dr. Griswold somewhat overestimated the dangers attending these applications. One case he referred to briefly, since it conveyed an important lesson. Some years ago he saw with Dr. I. B. Read and several other physicians, a case of what afterward proved to be opium-poisoning. The respiration was only 10 and the pulse 40. On applying the faradic current the respirations immediately rose to 18 and the pulse to 70, but whenever the applications were withheld for a short time both respiration and pulse immediately fell to 10 and 40 respectively. For two hours, by means of electric excitation, the respiration and pulse were upheld, but at the end of that time they began to fail, notwithstanding their best efforts. At the end of four or five hours both respiration and pulse were so infrequent and feeble that the case was regarded as hopeless and abandoned, and on the following day he was surprised to learn that soon after the cessation of treatment the patient began to rally, and finally recovered. It was sufficiently evident that the patient had been kept alive for hours by the efforts made. The electricity had simply tided her over the hours when the poison was exerting its most depressing influence. It was a mistake to have abandoned the case when they did, and it should suggest to every practitioner that such cases should not be given up while the slightest evidence of life remains.

Dr. Fordyce Barker directed attention to collateral points, with special reference to the effects produced by chloroform and ether, as illustrated in obstetrics. The general conclusion which he had long ago reached was that ether was much safer than chloroform in surgical practice where the anæsthetic was administered to prepare the patient for an operation, whereas, chloroform was much safer than ether in obstetric practice where the anæsthetic was given to relieve pain already present. Furthermore, the early use of chloroform in obstetric cases was indicated rather than contraindicated, when the woman had organic cardiac disease, contrary to the opinion that anæsthetics were not safe where cardiac disease existed. Dr. Barker had held this view and had carried it into practice during the last twenty-five years. But he would not use ether under the same circumstances.

Dr. R. W. Amidon wished to commend the remarks made by Dr. Griswold, and had nothing to offer in the way of criticism. He thought the paper was of the order which should receive the special commendation of the Academy, and made a motion to that effect, which was adopted.

Dr. Putzel thought that all the physiological deductions set forth in the paper of the evening were well put, but that the clinical inferences were not in accord with established clinical facts. The difficulty with most experiments on animals was that they could not be applied to the human body. He knew of no way in which the pneumogastrics could be galvanized in the human subject, aside from a surgical operation, because the current when applied to the surface diffused itself to an extent which did not permit us to speak of galvanization of the pneumogastrics; indeed, the best galvano-therapeutists employed the term galvanization of the neck.

He had applied electricity from twenty good elements, the number which the author of the paper had regarded as almost certain to produce death, without producing any bad effect whatever, and in exophthalmic goitre without producing any change in the condition of the pulse. From the clinical standpoint, the subject was one which required revision, and the truth could be reached only by prolonged observation of cases.

It would not do to accept cases in the report of which it was simply said that "electricity was applied," as something should be known concerning the size of the electrodes, strength and kind of current used, etc.

Dr. J. Leonard Corning referred to cases of exophthalmic goitre, in which galvanization of the pneumogastrics lowered the pulse-rate very markedly.

Dr. Putzel did not wish to be understood as saying that galvanization of the neck never produced slowing of the pulse, but simply that he had not observed that effect. He wished merely to state that galvanization of the neck was not galvanization of the pneumogastrics.

Dr. A. H. Smith thought that the object in view in the use of electricity in opium-poisoning was to stimulate respiration. This could be done by applying one pole over the phrenic nerve in the neck, and probably all the benefit which could be derived would follow the application of the current about sixteen or eighteen times a minute, and no more, and at the same time the dangerous depressing effect would be avoided. In the meantime, electricity could be used as a general stimulant, as was flagellation, etc.

The President referred to the use of electricity in the treatment of the asphyxia of the new-born. He had found it to be a valuable remedy in those cases, and had employed it as follows: When applied for four or five seconds in succession he had noticed that at the beginning the pulse and respiration were improved, to be followed by almost immediate paralysis. He had also found that this effect was produced the more surely when the application of the electrodes was confined to the region of the phrenic and pneumogastric nerves.

He therefore concluded, that the most practical way to use electricity was to apply it generally to the muscles, quickly striking the surface at different points with the electrodes. In opium-poisoning electricity was most effectual when applied at short intervals and by giving sudden shocks. At the same time, it should not be overlooked that in many cases cardiac failure was merely secondary to respiratory failure, and that when the respiration was improved by exciting the respiratory muscles, there would be a decided improvement in the action of the heart.

Dr. Griswold, in closing the discussion, reviewed the points made prominent in his paper, and said that he should prefer artificial respiration to general stimulation of the surface in the classes of cases under consideration. In chloroform cases, and those in which morphine had entered a vein, he would not use electricity at all, but would rely upon hypodermic injection of stimulants, inhalation of ammonia, and artificial respiration.

Dr. M. Josiah Roberts then gave a description and demonstration of the working of his perfected electro-osteotome, with a new form of portable storage battery, and an electrical illuminating apparatus. The description of his first instrument was published in the *Record*, October 27, 1883. To further aid the surgeon in applying the instrument when surrounded by insufficient light, he recommended Jarvis's electrical light attached to a head mirror.

The communication was discussed by Drs. Poore, Jarvis, Corning, Birdsall, Holcombe, and Fanning. The Academy then adjourned.

